# Jejunal Diverticulosis as a Rare Cause of Severe Obscure Gastrointestinal Bleeding in an Elderly Patient: A Case Report

**DOI:** 10.1002/ccr3.72826

**Published:** 2026-06-06

**Authors:** Mahmoud Draidi, Munir Abu Ageila, Arwa Ali, Mohanad Ibrahim, Abdelatif Abdelmola, Mohammed Danjuma

**Affiliations:** ^1^ Department of Internal Medicine Hamad Medical Corporation Doha Qatar; ^2^ Department of Gastroenterology Hamad Medical Corporation Doha Qatar; ^3^ Department of General Surgery Hamad Medical Corporation Doha Qatar

**Keywords:** capsule endoscopy, endoscopy, jejunal diverticulosis, laparoscopic surgery, obscure gastrointestinal bleeding, small bowel hemorrhage, surgical resection

## Abstract

Jejunal diverticulosis is a rare cause of obscure gastrointestinal bleeding (OGIB) that can present with life‐threatening hemorrhage. We report a 69‐year‐old male with recurrent melena, severe anemia, and hemodynamic instability requiring multiple transfusions. Upper and lower endoscopy were nondiagnostic, and CT angiography failed to localize the bleeding source. Capsule endoscopy showed distal small bowel bleeding without clear localization. The patient developed transfusion‐dependent bleeding complicated by *Klebsiella pneumoniae* sepsis. Due to persistent hemorrhage, diagnostic laparoscopy was performed and revealed two jejunal diverticula with engorged mesenteric vessels. Segmental small bowel resection was done with complete resolution of bleeding. This case highlights the limitations of noninvasive modalities in intermittent small bowel bleeding and supports early surgical intervention when the bleeding source cannot be identified.

## Key Clinical Message

Jejunal diverticulosis can cause life‐threatening obscure gastrointestinal bleeding that eludes conventional endoscopy, imaging, and capsule studies; in transfusion‐dependent patients with no localized source, early surgical exploration is both the diagnostic and definitive therapeutic step.

**Abbreviations**
AI: Artificial intelligence
ANC: Absolute neutrophil count
CRP: C‐reactive protein
ED: Emergency Department
EGD: Esophagogastroduodenoscopy
MICU: Medical Intensive Care Unit
NSAIDs: Nonsteroidal anti‐inflammatory drugs
OGIB: Obscure gastrointestinal bleeding
PPI: Proton pump inhibitor
PRBCs: Packed red blood cells
TIBC: Total iron‐binding capacity

## 1 Introduction

Obscure gastrointestinal bleeding (OGIB) is defined as recurrent or persistent gastrointestinal bleeding for which the source remains unidentified after negative upper and lower endoscopy. It accounts for approximately 5%–10% of all gastrointestinal bleeding episodes and represents a significant diagnostic and therapeutic challenge [1, 2]. This is exemplified in our patient, whose persistent and transfusion‐dependent bleeding remained unexplained despite extensive standard evaluation. The small bowel is the most frequent source of OGIB, but its evaluation is often difficult due to limited accessibility and intermittent bleeding [2].

Jejunal diverticula are an uncommon cause of small bowel bleeding, with a reported prevalence of 0.3%–2% in the general population. Most cases are asymptomatic and discovered incidentally, but they can occasionally lead to severe complications such as bleeding, diverticulitis, or perforation. Risk factors for developing jejunal diverticulosis include advanced age and comorbid conditions such as diabetes and hypertension [3, 4].

The diagnostic evaluation of OGIB has evolved significantly with the advent of capsule endoscopy, which allows direct visualization of the small bowel and has become the first‐line tool in assessing unexplained bleeding. Capsule endoscopy improves detection of mucosal lesions, vascular abnormalities, and small bowel tumors that may be missed on conventional endoscopy. In patients with jejunal diverticula or other small bowel sources, early identification is critical to guide appropriate therapeutic interventions and prevent recurrent hemorrhage [5].

## 2 Case Presentation

### 2.1 Case History/Examination

A 69‐year‐old Libyan male, a retired pilot, with a past medical history of hypertension controlled on enalapril 20 mg daily, type 2 diabetes mellitus (most recent HbA1c 6.7%) managed with sitagliptin‐metformin 50/1000 mg twice daily, dapagliflozin 10 mg once daily, glimepiride 4 mg twice daily, and insulin glargine 20 units at bedtime, and dyslipidemia controlled on rosuvastatin 10 mg nightly, presented to the emergency department with a 4‐day history of melena. He described the stools as black, tarry, and moderate in amount, occurring once to twice daily. He also reported dizziness, palpitations, and exertional shortness of breath beginning 2 days before admission. He endorsed mild, dull lower abdominal pain but denied hematemesis, hematochezia, nausea, vomiting, fever, or weight loss.

He had a history of *Helicobacter pylori* infection treated 10 years earlier and had undergone laparoscopic left inguinal hernia repair in May 2025. Postoperatively, he was prescribed celecoxib, which he took daily for 2 weeks while continuing aspirin 100 mg daily for primary cardiovascular prophylaxis. He was a long‐term shisha smoker and denied alcohol consumption.

On admission, the patient appeared pale and dehydrated. Vital signs revealed tachycardia (heart rate 105–120 bpm), borderline systolic blood pressure (90–100 mmHg), and oxygen saturation of 96% on room air. Physical examination demonstrated conjunctival pallor, dry mucous membranes, and melena on digital rectal examination. There were no stigmata of chronic liver disease.

### 2.2 Investigations

Laboratory investigations revealed severe anemia with hemoglobin 6.2 g/dL. Iron studies showed low‐normal serum iron (10 μmol/L), ferritin 39.4 μg/L, total iron‐binding capacity 53 μmol/L, and transferrin 2.1 g/L—findings consistent with iron‐deficiency anemia secondary to gastrointestinal blood loss. Other significant laboratory results are summarized in Table 1. The patient was resuscitated with intravenous fluids, transfused with packed red blood cells (PRBCs), and started on a continuous intravenous proton pump inhibitor (PPI) infusion. Throughout this period, he remained hemodynamically stable.

**TABLE 1:** First ICU admission was for massive gastrointestinal bleeding with hemodynamic instability; second ICU admission was for *Klebsiella pneumoniae* sepsis with septic shock.

Test	Hospital admission	First ICU admission	Second ICU admission	Hospital discharge	Reference range
WBCs (×10^3^/μL)	9.6	15.2	20.4	6.6	4–10
Hgb (g/dL)	6.2	5.6	7.7	9.5	13–17
Hematocrit (%)	18	16.2	23.6	29.4	40–50
Platelets (×10^3^/μL)	201	137	178	316	150–400
INR	1.2	1.2	—	1.2	—
APTT (S)	21.9	21.9	—	27	25–36.5
Creatinine (μmol/L)	119	66	74	55	70–115
Urea (mmol/L)	21.3	14.2	4	2.3	2.8–8.1
Sodium (mmol/L)	132	135	135	139	135–145
CRP (mg/L)	20.2	—	103.6	20.2	0–5
Lactic acid (mmol/L)	2.90	5.10	2	0.60	0.5–2.2

**FIGURE 1:**
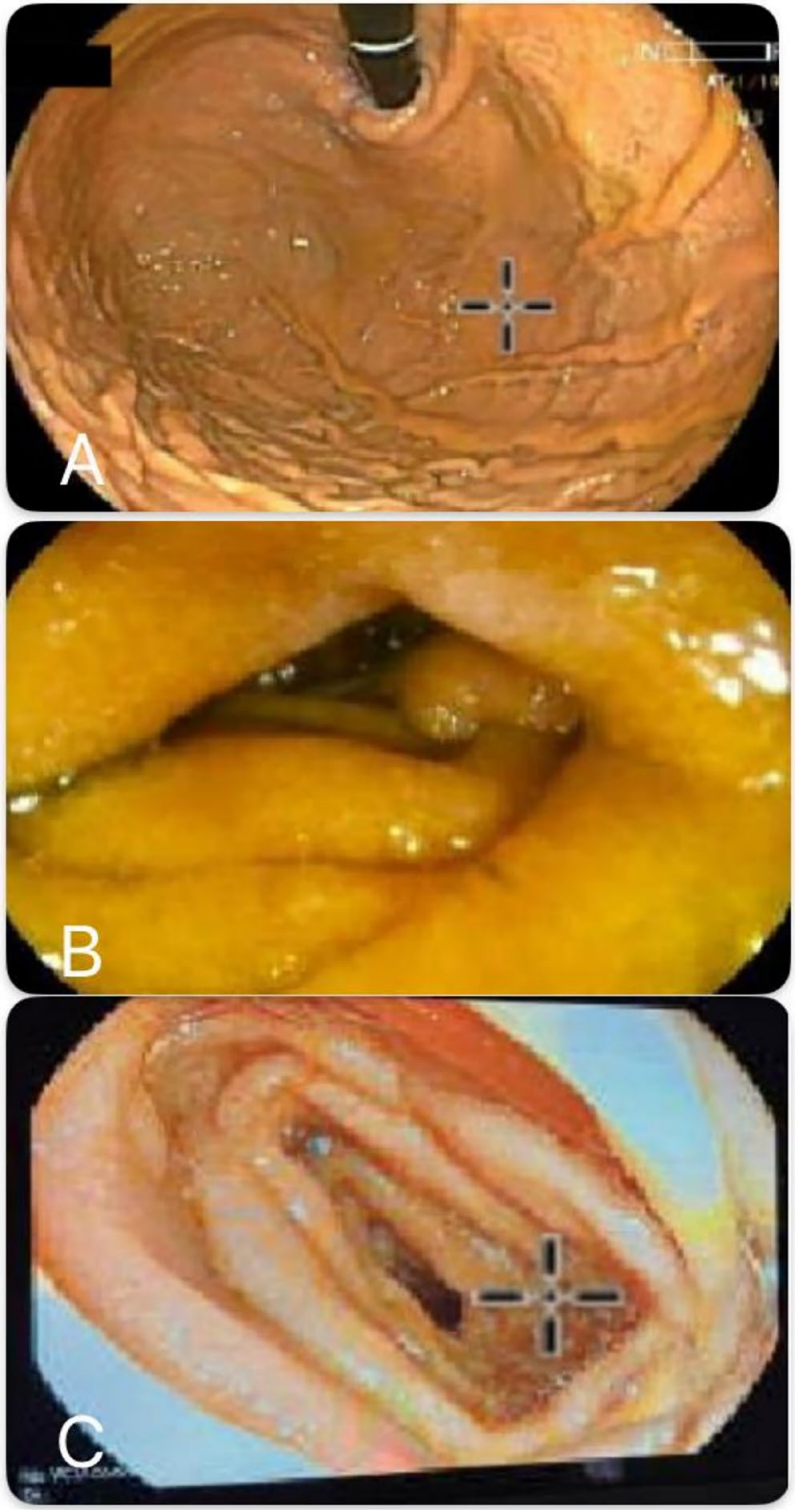
Upper endoscopy with push enteroscopy showing normal gastric mucosa (A), normal duodenum D3 (B), and normal proximal jejunum (C).

The first esophagogastroduodenoscopy (EGD) revealed mild antral erythema with erosions and a small hiatal hernia. The duodenal bulb showed edematous mucosa with prominent Brunner's glands. No ulcers, varices, polyps, or stigmata of recent hemorrhage were identified, and the CLO test was negative (Figure 1).

Subsequently, the patient developed acute massive gastrointestinal bleeding, presenting with melena and passage of fresh blood mixed with stool. This episode was associated with hypotension (BP 80/60 mmHg), tachycardia (HR 140 bpm), a hemoglobin drop to 5.6 g/dL, and venous lactate 5.1 mmol/L. He was transfused with additional PRBCs, fresh frozen plasma, and calcium chloride and transferred to the medical intensive care unit (MICU).

CT angiography of the abdomen and pelvis demonstrated subtle hyperdensity in the pyloric region during the venous phase and a faint contrast blush in the rectum, but no definitive arterial extravasation. Incidental small colonic diverticula were seen without evidence of diverticulitis or pericolic collection. Interventional radiology deferred embolization due to the absence of active bleeding.

After stabilization, the patient was transferred to the medical ward. Approximately 1 week later, he developed sepsis secondary to Gram‐negative bacteremia. Blood cultures grew *Klebsiella pneumoniae* . No clear infectious source was identified at the time, and the bacteremia was considered most likely secondary to gut translocation in the context of ongoing severe gastrointestinal bleeding and possible bowel mucosal compromise. He was started on piperacillin‐tazobactam and vasopressor support with noradrenaline (0.15 μg/kg/min) and was returned to the MICU. A repeat abdominal CT scan redemonstrated scattered colonic diverticula with foci of calcification but no active bleeding or free intraperitoneal fluid.

**FIGURE 2:**
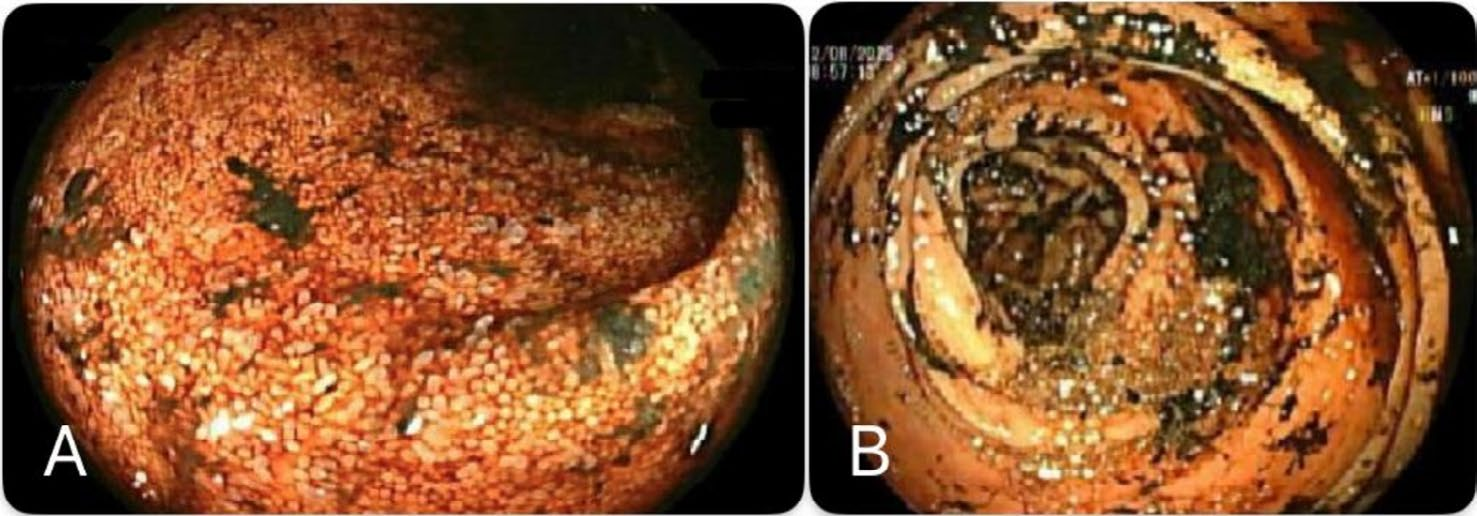
Colonoscopy showing altered blood and melanotic stools in the terminal ileum (A) and ascending colon (B).

During his MICU stay, the patient experienced multiple further episodes of melena and passage of fresh blood mixed with stool, associated with recurrent drops in hemoglobin, necessitating several endoscopic evaluations. The first colonoscopy revealed a lumen filled with melena that limited visualization; after lavage, no active bleeding, ulcers, or vascular lesions were detected. The second colonoscopy advanced to the terminal ileum, demonstrating normal mucosa throughout with medium‐sized internal hemorrhoids but no stigmata of recent bleeding. A third colonoscopy later performed from the anus to the terminal ileum again revealed altered blood and melanotic stools throughout, raising suspicion for a small bowel or upper gastrointestinal source as shown in Figure 2.

Then the plan was to further investigate possible upper GI bleeding source, so a repeat upper endoscopy with push enteroscopy using a pediatric scope was advanced into the proximal jejunum. The mucosa appeared grossly normal, and mild antral erosive gastritis was again noted.

**FIGURE 3:**
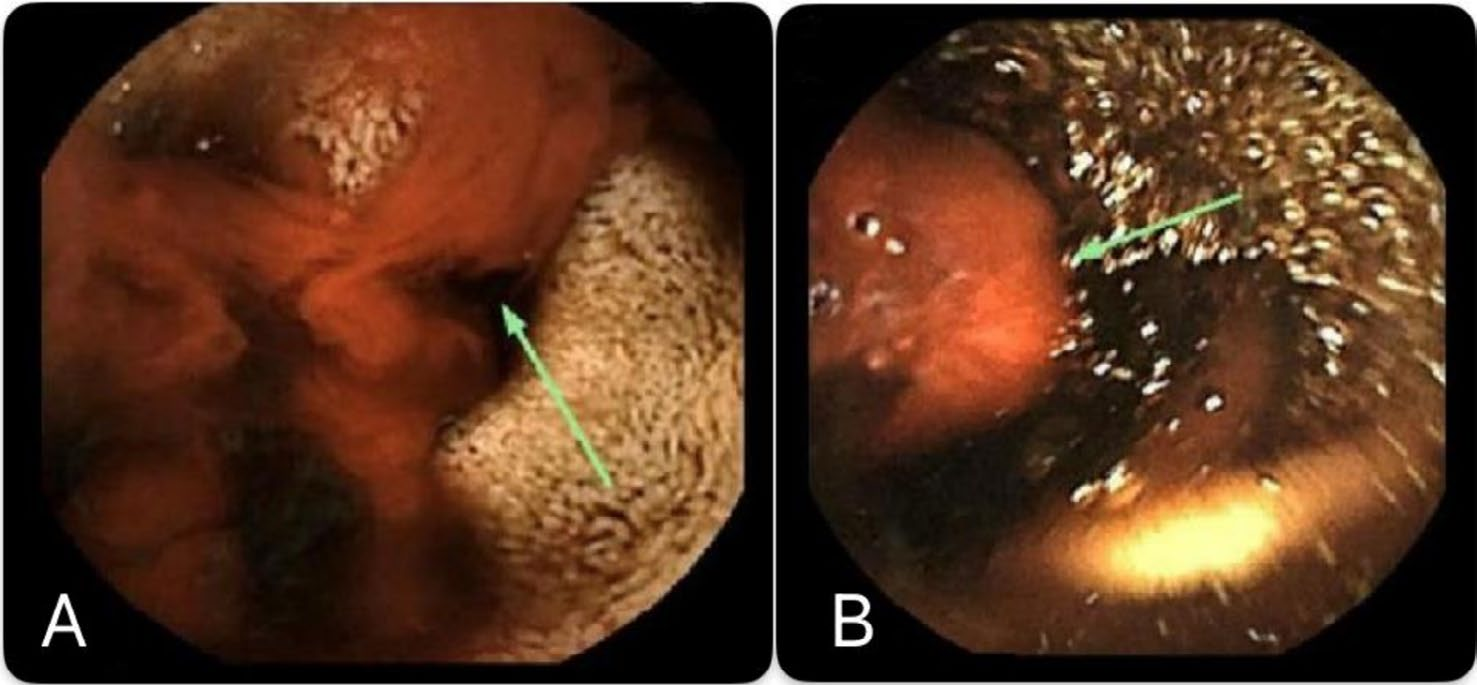
Video capsule endoscopy showing altered blood (A) and organized clot (B) with fresh blood seen mainly at the ileum.

Then video capsule endoscopy was performed which demonstrated strong evidence of small bowel bleeding, showing altered blood, organized clots, and fresh blood predominantly in the distal small bowel as shown in Figure 3. Technetium‐99 m pertechnetate (Meckel's) scan was negative for Meckel's diverticulum.

### 2.3 Treatment

During the first 2 weeks of hospitalization, the patient received a total of eight units of PRBCs to maintain hemoglobin above 8 g/dL due to ongoing obscure gastrointestinal bleeding. Given persistent bleeding and inconclusive findings on noninvasive investigations, the upper gastrointestinal surgery team proceeded with diagnostic laparoscopy.

**FIGURE 4:**
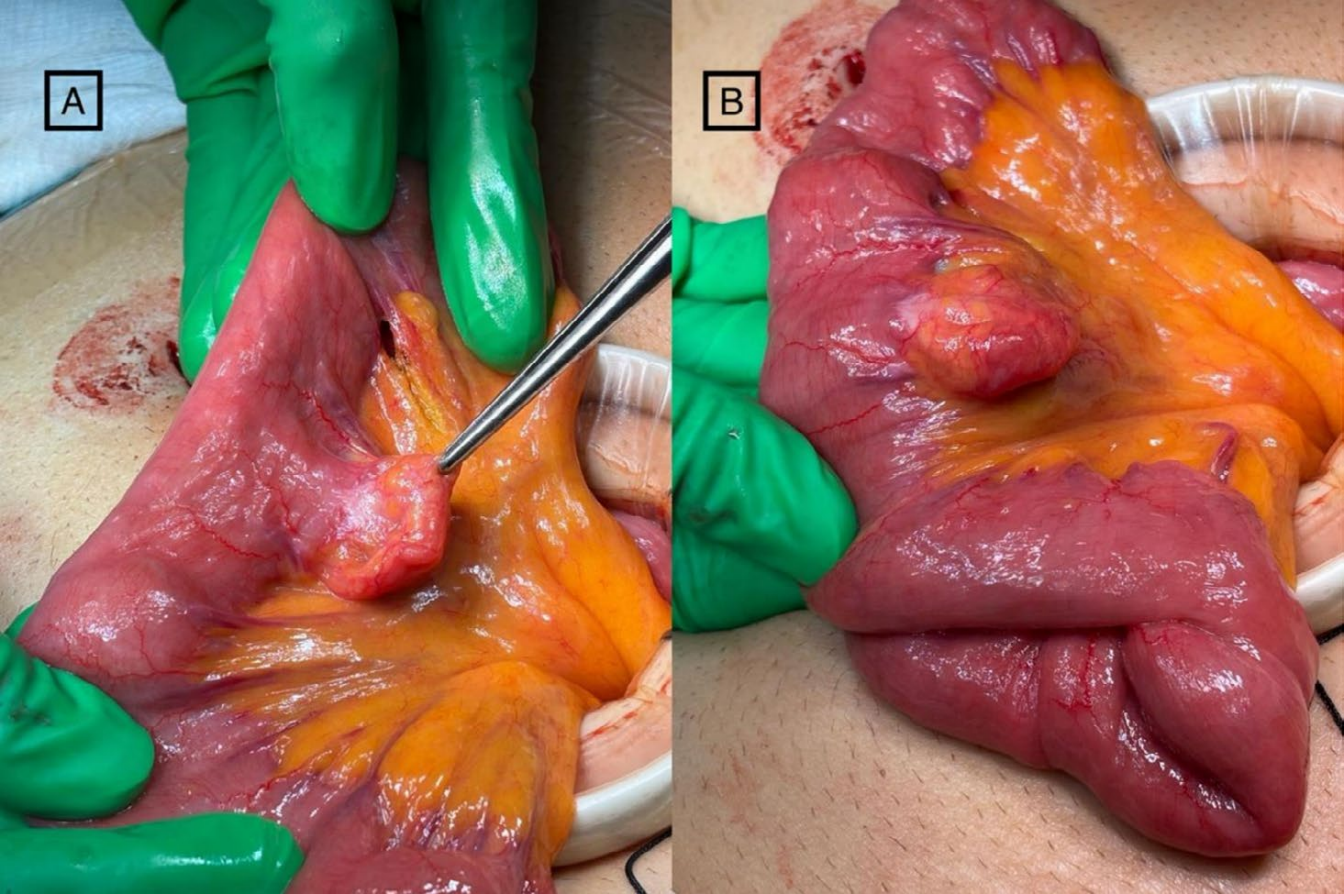
Intraoperative images of the jejunal lesion after exteriorization through mini laparotomy wound. (A) The diverticulum is seen as a saccular outpouching on the mesenteric border of the jejunum, with intact serosa and adjacent engorged mesenteric vessels. (B) A different angle of the same lesion shows its broad base. No evidence of perforation, ischemia, or contamination is observed.

**FIGURE 5:**
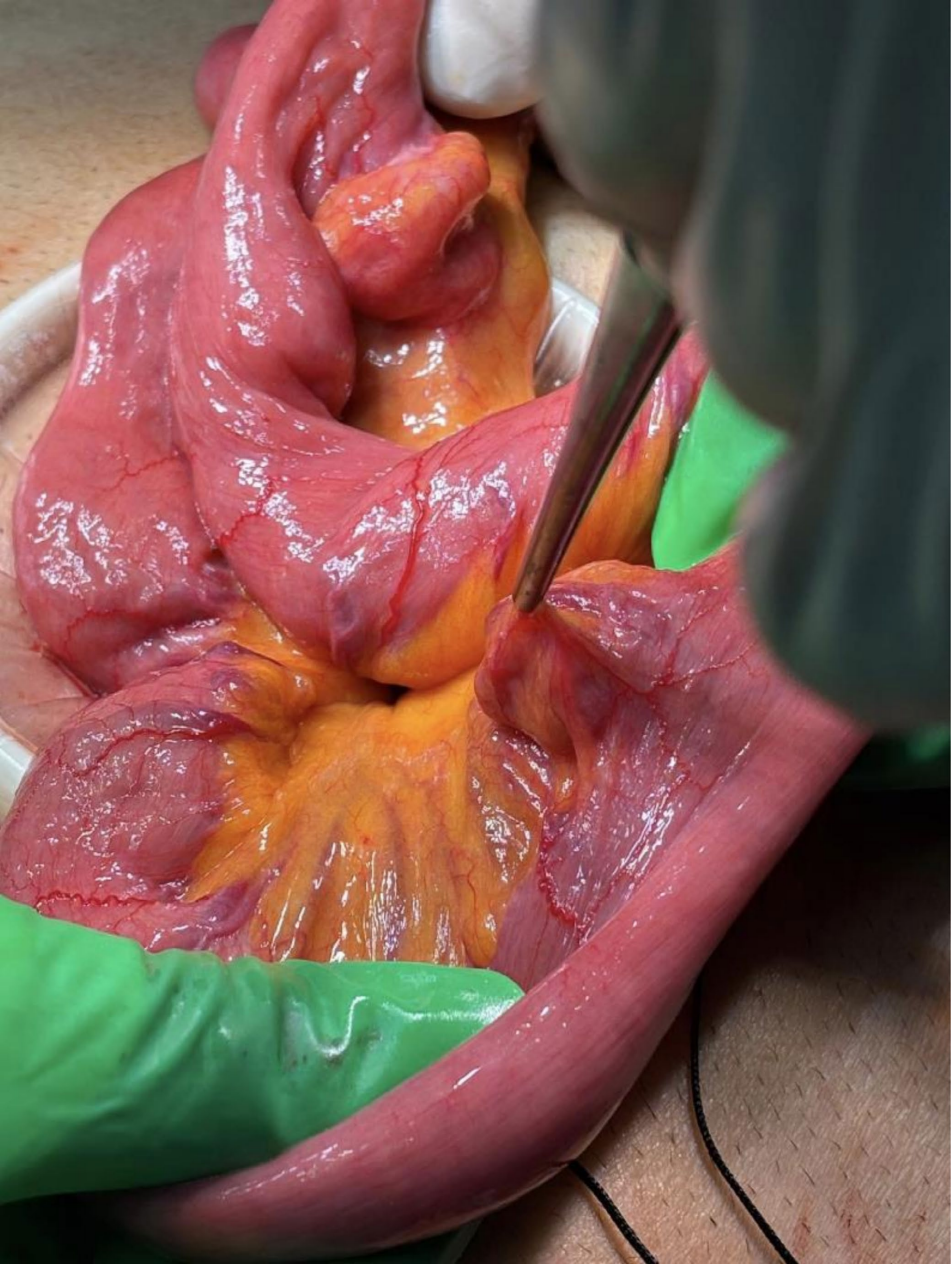
An intraoperative image demonstrates a second jejunal diverticulum distal to the previous one. Exhibiting a prominent engorged vessel along its wall, consistent with the findings observed in the first diverticulum.

Intraoperatively, the small bowel was run from the duodenojejunal (DJ) flexure to the ileocecal valve (ICV). Two jejunal diverticula with associated adjacent engorged mesenteric vessels were identified, located approximately 90 cm and 110 cm distal to the DJ flexure. No other pathology was noted (Figures 4 and 5).

A mini laparotomy was performed through a supra‐umbilical incision, and a wound protector was placed. The affected segment of small bowel was exteriorized and approximately 40 cm of small bowel was resected (including the two diverticula). Then side‐to‐side small bowel anastomosis was created using a 75 mm linear stapler (Ethicon). The enterotomy was closed transversely in two layers using 3/0 V‐Loc sutures. The mesenteric defect was subsequently closed with absorbable sutures.

Intraoperative enteroscopy was not performed, as no other pathology was identified on thorough inspection. The diverticulas were considered the most likely source of bleeding based on preoperative capsule endoscopy findings and the presence of adjacent engorged mesenteric vessels. No active bleeding was observed intraoperatively; therefore, the attribution was presumptive but supported by the clinical and intraoperative finding.

Postoperative recovery was uneventful, with complete resolution of gastrointestinal bleeding and stable hemoglobin levels without further transfusion requirements. He was discharged home in good condition and, at 1‐month follow‐up, remained asymptomatic with no recurrence of melena and normal hemoglobin. A detailed chronological summary of the patient's clinical course is presented in Table 2.

**TABLE 2:** Chronological summary of clinical course, investigations, complications, and management during hospital admission.

Time point	Event	Key clinical findings/interventions
Hospital presentation	Initial presentation with melena	4‐day history of melena with dizziness, palpitations, and exertional dyspnoea. Pale, tachycardic, borderline hypotensive. Hb 6.2 g/dL → acute gastrointestinal bleeding with severe iron‐deficiency anemia Resuscitation with IV fluids, PRBC transfusion, and IV PPI initiated
Early hospital course	Hemorrhagic shock episode	Hemodynamic instability (BP 80/60 mmHg, HR 140 bpm), fall in Hb to 5.6 g/dL, lactate 5.1 mmol/L → massive transfusion protocol activated and ICU admission. Initial EGD and CT angiography were negative for bleeding source
ICU admission (hemorrhagic phase)	Persistent obscure GI bleeding	Ongoing melena and fresh blood per rectum requiring repeated transfusions and intensive care monitoring
ICU readmission (sepsis episode)	Infectious complication	Development of *Klebsiella pneumoniae* bacteraemia → treated with piperacillin‐tazobactam and vasopressor support (noradrenaline). ICU readmission for septic shock management
Diagnostic workup phase	Comprehensive evaluation for OGIB	Multiple colonoscopies (to terminal ileum) negative; push enteroscopy negative; capsule endoscopy showed fresh blood and clots in distal small bowel; Meckel's scan negative
Operative intervention	Surgical exploration	Diagnostic laparoscopy revealed two jejunal diverticula with adjacent engorged mesenteric vessels (~90 cm and 110 cm from DJ flexure). Segmental small bowel resection (40 cm) with primary anastomosis performed
Postoperative course	Recovery and outcome	Uneventful recovery with complete resolution of bleeding. Stable hemoglobin without transfusion. Asymptomatic at 1‐month follow‐up with normal Hb and no recurrence of melena

Abbreviations: Hb, hemoglobin; ICU, intensive care unit; OGIB, obscure gastrointestinal bleeding; PRBC, packed red blood cells.

## 3 Discussion

OGIB remains a significant diagnostic and therapeutic challenge, accounting for 5%–10% of all gastrointestinal bleeding cases [1]. Defined as recurrent or persistent bleeding after negative upper and lower endoscopy, the small bowel is the most frequent source [2]. Within this segment, jejunal diverticula are a rare etiology, occurring in < 2% of the population and predominantly affecting elderly males. Although often asymptomatic, they may present with OGIB or, less frequently, obstruction and perforation, posing a significant diagnostic and management challenge. In addition to bleeding, jejunal diverticulosis may present with inflammatory complications such as diverticulitis, which can mimic inflammatory bowel disease (IBD) clinically and radiologically, leading to potential misdiagnosis and delay in appropriate management. Their nonspecific symptoms and intermittent bleeding patterns often lead to delayed recognition and management [6, 7, 8].

Our patient presented with overt OGIB characterized by recurrent melena, symptomatic anemia, and multiple transfusion requirements. Despite extensive evaluation—including bidirectional endoscopy, capsule endoscopy, and radiologic studies—the bleeding source remained elusive. This underscores a major limitation of conventional modalities in OGIB, particularly for intermittent bleeding from subtle sources like jejunal diverticula [5, 9]. Capsule endoscopy, while first‐line for small bowel assessment, has variable sensitivity (38%–83%), influenced by lesion type, bleeding timing, and bowel preparation. Blood or intestinal contents can obscure lesions, especially small vascular or mucosal defects [10, 11]. In our patient, capsule endoscopy revealed the presence of fresh blood and clots within the jejunal loops, suggesting recent hemorrhage; however, the precise bleeding source could not be localized. This may be explained by rapid luminal transit, obscuration of the mucosa by blood, and the presence of multiple diverticula, all of which can hinder accurate identification of the exact bleeding site. Such findings illustrate the diagnostic challenge posed by jejunal diverticulosis, where intermittent or self‐limited bleeding may occur between imaging intervals, resulting in nondiagnostic studies.

The episode of Gram‐negative bacteremia during hospitalization added further complexity to the clinical course. Although no definitive infectious source was identified on imaging, *Klebsiella pneumoniae* bacteremia in the setting of ongoing severe gastrointestinal bleeding raises the possibility of bacterial translocation across a compromised intestinal mucosa. In retrospect, and following surgical confirmation of jejunal diverticulosis, this may reflect underlying structural bowel pathology predisposing to mucosal vulnerability and impaired barrier function. However, this association remains speculative, and the primary clinical implication of this episode was the need for stabilization prior to definitive diagnostic surgical exploration.

The pathophysiology of jejunal diverticular bleeding is multifactorial. Diverticula arise from herniation of mucosa and submucosa through weak points in the muscular wall at sites where mesenteric vessels penetrate. These areas are predisposed to mucosal ischemia, ulceration, and vascular erosion. Risk factors include advanced age, motility, and coagulation disorders. In our case, the patient had been taking chronic low‐dose aspirin and received celecoxib postoperatively. Although celecoxib is a selective COX‐2 inhibitor with a lower risk of gastrointestinal toxicity, this protective effect is significantly reduced when combined with aspirin, potentially increasing mucosal vulnerability and contributing to the bleeding episode. Diverticula may coexist with angiodysplasias or ulcers, further increasing hemorrhage risk. While conservative measures or endoscopic therapy may suffice in select cases, recurrent or hemodynamically significant bleeding often necessitates surgical resection [12, 13, 14, 15].

Device‐assisted enteroscopy, including double‐ and single‐balloon techniques, allows deep small bowel intubation for both diagnostic and therapeutic purposes. These procedures can identify bleeding sources missed by conventional endoscopy and permit endoscopic interventions such as clipping or coagulation [16, 17]. Interventions include argon plasma coagulation, clipping, or injection therapy. These procedures, however, are technically demanding, limited in availability, and may not be feasible in acutely unstable patients. Consequently, surgery remains the definitive treatment for patients with refractory or massive bleeding [18]. In our case, device‐assisted enteroscopy was not available at our institution, limiting further small bowel evaluation and necessitating surgical exploration for definitive management.

Radiologic imaging is a critical adjunct in OGIB. CT enterography provides detailed mucosal and extramucosal assessment, while CT angiography rapidly localizes active bleeding and guides therapy. In our patient, CT angiography failed to localize a definitive bleeding source despite clinical evidence of ongoing hemorrhage. This is most likely explained by the intermittent nature of bleeding from jejunal diverticula, resulting in the absence of active arterial extravasation at the time of imaging. The subtle venous‐phase findings (pyloric hyperdensity and faint rectal blush) were nonspecific and did not guide intervention. This highlights a key limitation of CT angiography, which relies on active bleeding at a sufficient rate for detection and may therefore be falsely negative in cases of intermittent small bowel hemorrhage such as in our case. Angiographic embolization offers a minimally invasive alternative in high‐risk surgical candidates, though its success depends on active bleeding and carries a risk of ischemia [19, 20].

Surgical management of jejunal diverticular bleeding, particularly segmental resection, is associated with favorable outcomes and lower recurrence compared with limited diverticulectomy. Timely surgery is indicated in patients with massive hemorrhage, repeated transfusion requirements, or failure of endoscopic and radiologic therapies. In our patient, the decision to proceed with surgical exploration was driven by persistent transfusion‐dependent bleeding, hemodynamic instability, and failure of endoscopic and radiologic modalities to localize the source. This underscores the importance of early surgical consideration in similar cases where ongoing bleeding poses a life‐threatening risk and noninvasive diagnostics remain inconclusive [13].

## 4 Conclusion

Jejunal diverticulosis is a rare and often under‐recognized cause of obscure gastrointestinal bleeding, particularly in elderly patients. Diagnosis can be challenging due to intermittent bleeding and limitations of endoscopy and imaging. A multimodal approach, including capsule endoscopy and timely surgical intervention when indicated, ensures definitive management and prevents recurrence.

## Author Contributions

**Mahmoud Draidi:** conceptualization, data curation, writing – original draft. **Munir Abu Ageila:** writing – original draft. **Arwa Ali:** writing – original draft. **Mohanad Ibrahim:** writing – original draft. **Abdelatif Abdelmola:** writing – review and editing. **Mohammed Danjuma:** writing – review and editing.

## Funding

Open Access funding provided by the Qatar National Library.

## Ethics Statement

The article was approved by the Institution Review Board at Hamad Medical Corporation.

## Consent

A written informed consent was obtained from the patient to publish all images, clinical data, and other data included in the manuscript. All identifying information has been removed.

## Conflicts of Interest

The authors declare no conflicts of interest.

## Data Availability

The data supporting this case report are not publicly accessible due to patient confidentiality but may be made available by the corresponding author upon reasonable request.
